# Photosymbiosis reduces the environmental stress response under a heat challenge in a facultatively symbiotic coral

**DOI:** 10.1038/s41598-024-66057-2

**Published:** 2024-07-05

**Authors:** D. M. Wuitchik, H. E. Aichelman, K. F. Atherton, C. M. Brown, X. Chen, L. DiRoberts, G. E. Pelose, C. A. Tramonte, S. W. Davies

**Affiliations:** 1 Department of Biology, Boston University, Boston, MA USA; 2 Department of Biology, Tufts University, Medford, MA USA; 3 Bioinformatics Graduate Program, Boston University, Boston, MA USA; 4 Department of Biology, Boston College, Boston, MA USA

**Keywords:** Symbiosis, Coral, Climate change, Environmental stress, Ecology, Ecology

## Abstract

The symbiosis between corals and dinoflagellates of the family Symbiodiniaceae is sensitive to environmental stress. The oxidative bleaching hypothesis posits that extreme temperatures lead to accumulation of photobiont-derived reactive oxygen species ROS, which exacerbates the coral environmental stress response (ESR). To understand how photosymbiosis modulates coral ESRs, these responses must be explored in hosts in and out of symbiosis. We leveraged the facultatively symbiotic coral *Astrangia poculata*, which offers an opportunity to uncouple the ESR across its two symbiotic phenotypes (brown, white). Colonies of both symbiotic phenotypes were exposed to three temperature treatments for 15 days: (i) control (static 18 °C), (ii) heat challenge (increasing from 18 to 30 °C), and (iii) cold challenge (decreasing from 18 to 4 °C) after which host gene expression was profiled. Cold challenged corals elicited widespread differential expression, however, there were no differences between symbiotic phenotypes. In contrast, brown colonies exhibited greater gene expression plasticity under heat challenge, including enrichment of cell cycle pathways involved in controlling photobiont growth. While this plasticity was greater, the genes driving this plasticity were not associated with an amplified environmental stress response (ESR) and instead showed patterns of a dampened ESR under heat challenge. This provides nuance to the oxidative bleaching hypothesis and suggests that, at least during the early onset of bleaching, photobionts reduce the host’s ESR under elevated temperatures in *A. poculata*.

## Introduction

The photosymbiosis between coral hosts and endosymbiotic algae in the family Symbiodiniaceae forms the backbone of entire coral reef ecosystems. This symbiosis is particularly important as tropical reef-building corals are found in nutrient poor waters, which are often carbon and nitrogen limited^1^. Much of the organic carbon required by coral hosts comes from translocation of photosynthetically derived sources from the symbiotic algae (photobiont)^2^ and hosts can actively promote photosynthesis through acidification of the symbiosome where photobionts reside^3^. Nitrogen limitation is overcome in part through interactions between the host and photobiont that conserve and recycle nitrogen between partners^4^. Coral-algal photosymbiosis is therefore vital to a healthy coral reef ecosystem; however, this relationship is vulnerable to rising seawater temperatures associated with anthropogenic climate change, and warmer temperatures lead to the breakdown of photosymbiosis (i.e., dysbiosis) in a process termed coral bleaching^5,6^. Bleaching can lead to coral mortality as hosts are deprived of symbiont-derived carbon sugars^7^, and bleaching events have led to dramatic declines in coral reefs globally^8^, negatively impacting coastal communities^9,10^. While rising temperatures are the most immediate threat to coral reefs, cold water extremes also cause coral bleaching and pose significant thermal challenges to coral species that are rarely investigated alongside rising temperatures^11–13^. As coral bleaching episodes become more frequent and severe as climate change accelerates^14^, understanding the mechanisms underlying this dysbiosis has become increasingly important.

Coral bleaching research has largely focused on corals that exhibit obligate symbiotic relationships (for review, see^15^). These works have highlighted the importance of heat-shock proteins^16,17^, antioxidant pathways^18^, and immunity^19,20^ in the coral host environmental stress response (ESR). A recent meta-analysis of the transcriptomic stress responses in tropical *Acropora* corals compared gene expression profiles from 14 distinct stress experiments^21^. This work established a link between the magnitude of stress imposed on corals with the direction of enrichment of key gene ontology (GO) terms associated with environmental stress response (ESR). A positive or negative association with these key GO terms can be used to qualitatively assess whether an experimental stressor resulted in a high-intensity ESR (‘Type A’ response) or if corals experienced a muted stressor that elicited a less severe ESR (‘Type B’ response)^21^. These associations have since been used across other coral taxa to explore gene expression enrichment patterns, enabling a characterisation of the severity of stress response being experienced by the coral. For example, we previously showed that white *A. poculata* exhibited unique ESRs in response to different temperature challenges^22^. Aichelman et al*.*^23^ also observed that *Oculina arbuscula* under heat and cold challenges exhibited divergent responses; however they also demonstrated that these responses were not modulated by symbiotic phenotype. This comparative approach leveraging the meta-analysis by Dixon et al.^21^ allows for the contextualisation of ESRs in divergent coral taxa across a variety of stressors. These characterisations have served as a useful qualitative tool in ascertaining whether experimental treatments elicit similar ESRs across gene expression studies in corals^22^. While we now have a broad understanding of coral ESRs in obligate symbiotic hosts, these corals cannot survive without their photobionts for extended time periods due to nutritional constraints^2^. Therefore, disentangling the effects of temperature from those associated with nutritional stress when photosymbiosis is lost remains a challenge, leaving critical gaps in our understanding of how photosymbiosis modulates coral ESRs (but see^23^).

Photosymbiosis may alter the coral host ESR depending on the context of the stressor. One way that this can occur is through the transfer of reactive oxygen species (ROS) from the photobiont to the host. While ROS is a natural by-product of photosynthesis, temperature stress may cause dysfunction in the photosynthetic machinery, which can further amplify ROS production^24^. This accumulation of photobiont derived ROS is thought to be a key factor in coral bleaching, as ROS leakage to the host is posited to lead to cellular damage and initiation of bleaching related signal cascades. This is referred to as the oxidative bleaching hypothesis, which has been well supported with experimental evidence demonstrating that photobionts can impose oxidative stress on cnidarian hosts^24–26^. For example, ROS leakage from freshly isolated photobionts in a heat stress experiment can increase by up to 45%, correlating with increases in coral host oxidative damage^27^. This pattern of photoinhibition followed by ROS accumulation is not limited to heat stress and has been suggested for cold stress as well^24^. While photoinhibition is well supported prior to bleaching^28–30^, bleaching can also occur without apparent buildup of photobiont derived ROS^25^. Furthermore, hosts upregulate oxidative stress-response pathways even when photobionts are absent^31^, and in some circumstances expelled photobionts during bleaching that did not show evidence of photoinhibition^32^. Therefore, photo-oxidative stress may not always be the weak link leading to dysbiosis. Alternatively, photobionts may provide critical energy reserves necessary for the host’s ESR^33^. For example, production of heat shock proteins that aid in repairing cell damage is energetically costly^34^ and additional energetic reserves may mitigate bleaching. Indeed, heterotrophy can reduce the probability of dysbiosis^35,36^. Ultimately, to understand if photosymbiosis modulates the ESR and supports the oxidative bleaching hypothesis (Table 1), these responses must be explored in coral hosts in and out of symbiosis.

**Table 1:** Predictions based on the oxidative bleaching hypothesis (H_1_) in how photosymbiosis impacts the coral environmental stress response (ESR) during thermal challenges.

Oxidative bleaching hypothesis	H_0a_ No effect of photosymbiosis	H_0b_ Photosymbiosis decreases ESR	H_1_ Photobiont derived ROS increases ESR
Differential expression between symbiotic phenotypes under thermal challenge	No	Yes	Yes
Key expression signatures	NA	No change in oxidative stress under thermal challenge	Increase in oxidative stress in brown corals
ESR (Dixon et al*.* ^21^)	No association	Type B	Type A

H_0a_ represents the null hypothesis with no effect of photosymbiosis, and H_0b_ represents an alternative null hypothesis having a decrease in the ESR.

Coral-algal photosymbioses exist along a continuum, with some coral species being completely heterotrophic and others being fully reliant on autotrophy of their photobionts^37^. Facultatively symbiotic corals exist across this continuum, offering the opportunity to disentangle host responses in and out of symbiosis by leveraging the white and brown phenotypes, which occur naturally on subtropical and temperate reefs^38–43^. These phenotypes correspond to photobiont densities, with brown colonies having naturally higher photobiont densities than white colonies even when collected from the same microenvironment^38^. It is important to note that while a colony can appear white to an observer, this phenotype does not mean that these colonies are completely devoid of photobionts (aposymbiotic). Rather, the white phenotype refers to lower photobiont densities hosted by these colonies (white colonies < 1 × 10^5^/cm^2^), and these algae have been shown to have a *de minimis* physiological effect on the coral host^44^. Leveraging these different phenotypes of *A. poculata* has demonstrated that photosymbiosis increases coral growth^38,39,45^ and aids in the recovery after injury^11,46^. This could be due to additional nutritional input as well as nitrogen assimilation provided by the photobionts^42^. The impact of photosymbiosis is likely influenced by temperature, as cooler temperatures dampen the growth benefits of this relationship in *A. poculata*^47^ and can even influence the host’s thermal optima^48^. Leveraging these symbiotic phenotypes in facultative corals has been useful in exploring key molecular pathways of photosymbiosis maintenance, including nitrogen cycling^42^, symbiont density regulation^39,44^, and immunity^49^. The facultatively symbiotic coral, *Astrangia poculata,* has emerged as a model system for symbiosis research^42,50,51^ and previous gene expression work from our group found that white *A. poculata* exhibit classic ESRs consistent with those observed in tropical reef-building corals, suggesting that it serves as a strong model for coral bleaching research^22^.

Here, we build on this work by leveraging white and brown colonies of *A. poculata* to explore how symbiosis modulates the host’s ESR with the goal of testing the oxidative bleaching hypothesis. Under this hypothesis, we would expect an elevated ESR in brown corals due to the accumulation of photobiont derived ROS, and a more muted ESR in white hosts (Table 1). We exposed white and brown *A. poculata* to both cold and heat challenges and sampled for gene expression to examine molecular snapshots of how symbiosis modulates the host’s ESR. To gain deeper insights into these responses, we compare these ESR profiles with those observed in tropical reef-building corals. Together, these data provide insights into the molecular repertoires a facultative coral host in and out of symbiosis engages to withstand thermal challenges.

## Materials and methods

### *Astrangia* poculata thermal challenge experimental design

*Astrangia poculata* colonies (N = 20) were collected from Woods Hole, MA, USA (N41° 31.51, W70° 40.49; Fig. S1) and shipped overnight to Boston University. Each colony was cut into three fragments, attached to petri dishes using cyanoacrylate glue, and maintained at 18 °C for several months of recovery. Colonies were classified by phenotype as either white or brown and then randomly assigned to one of three treatments (Table S1). Each treatment consisted of three replicate experimental tanks with temperatures maintained by Aqualogic Digital Temperature Controllers connected with aquarium heaters and chillers. A 12:12 h light:dark cycle was maintained with an intensity of 50 µmol m^−2^ s^−1^ and fragment positions were systematically rotated throughout to avoid potential differences in light exposure and water flow. Temperature and salinity were monitored daily using a YSI pro30 multiprobe and these values were confirmed with a glass thermometer and refractometer. The experiment was run for 15 days, and control tanks were maintained at 18 °C (salinity 35.3 ± 0.6 ppt) for the duration of the experiment. Tanks in the cold challenge were cooled from 18 °C by approximately 1 °C per day until a final temperature of 4 °C was achieved (salinity 34.5 ± 1.5 ppt), and tanks in the heat challenge were heated from 18 °C by approximately 1 °C per day until a final temperature of 30 °C was reached (salinity 35.0 ± 1.1 ppt) (Fig. S1B,D). Hourly temperature data between 2014 and 2021 were obtained from the National Oceanic and Atmospheric Administration (NOAA) weather buoy number BZBM3 and plotted with experimental challenge temperatures to compare treatments relative to collection site temperatures (Fig. S1C).

### Coral behavioural response to food stimuli

To determine behavioural responses to temperature challenge and symbiotic phenotypes, coral polyp behaviours in response to food stimulus were quantified following previous studies^22,52^. In brief, polyp activity was scored daily on a scale of 1 to 5 based on the percentage of active polyps within a fragment (1 = 0%, 2 = 25%, 3 = 50%, 4 = 75%, 5 = 100%) 30 min after freeze-dried copepods (Argent Cyclop-Eeze) were suspended in seawater. The same observer conducted each assay to limit observer biases. A cumulative link mixed model for ordinal-scale observations using the R package *ordinal*^53^ was generated with genotype and experimental tanks as random effects, and temperature treatment, symbiotic phenotype, and experimental day as fixed effects.

### Tag Seq library preparation and sequencing

Upon reaching maximum differences between thermal challenge and control treatments (Day 15), polyps from replicate colonies (N_brown_ = 8, N_white_ = 8) were sampled from each treatment (N_total samples_ = 48) for gene expression profiling. Several polyps were removed from each fragment using sterilized bone cutters, immediately preserved in 200 proof ethanol, and stored at − 80 °C. Total RNA was extracted using an RNAqueous kit (Ambion by LifeTechnologies) following manufacturer’s recommendations. An additional step was implemented using 0.5 mm glass beads (BioSpec), which were added to the lysis buffer and samples were homogenized using a bead beater for 1 min. RNA quantity was determined using a DeNovix DS11+ spectrophotometer and integrity was assessed by visualising ribosomal RNA bands on 1% agarose gels. Trace DNA contamination was removed using a DNase 1 (Ambion) digestion at 37 °C for 45 min. Libraries were created from 1500 ng of total RNA following Meyer et al.^54^ and adapted for Illumina Hi-Seq sequencing^55^. In brief, RNA was heat sheared and transcribed into first strand cDNA using a template switching oligo and SMARTScribe reverse transcriptase (Clontech). cDNA was PCR amplified, individual libraries were normalized, and Illumina barcodes were incorporated using a secondary PCR. Samples were pooled and size-selected prior to sequencing on Illumina Hiseq 2500 (single-end 50 base pairs) at Tufts University Core Facility.

### Gene expression analyses

References for *A. poculata*^56^ and its homologous photobiont *Breviolum psygmophilum*^49^ were concatenated to form a holobiont reference. Raw sequence files were trimmed to remove adapters and poly-A tails using the *fastx-Toolkit* (v 0.0.14, fastx-Toolkit. http://hannonlab.cshl.edu/fastx_toolkit.) and sequences that were < 20 bp in length were removed. Sequences with > 90% of bases having a quality score > 20 were retained, PCR duplicates were removed, and resulting quality-filtered reads were mapped to the holobiont reference using *bowtie2* v2.4.2^57^. Samples maintained in analyses had an average of 703,688 (SD = 298,412) mapped reads and five individuals were removed due to low read depth (< 100,000/sample, Table S3). Of the remaining samples, mapping efficiencies ranged from 37 to 83% with an average mapping efficiency of 69% (SD = 9.4%) (Table S4). Reads that were assigned to the photobiont (1042-41035 total reads; 0.1–10.0%) were then discarded and only host reads were used for subsequent analyses.

The presence of clones in the dataset was checked by identifying single nucleotide polymorphisms (SNPs) across samples. Reads from each sample were mapped to the host genome using *bowtie* v2.4.2^57^, which produced sam alignment files that were converted to bam files using *sortConvert* in *samtools*^58^. An identity-by-state matrix was then generated using *ANGSD* v.0935^59^ with loci filtered to include those present in 78% of individuals, having a minimum quality score of 25 and a mapping score of 20. Further parameters in *ANGSD* were set so that a strand bias p-value > 1 × 10^−5^, minimum minor allele frequency > 0.05, p-value > 1 × 10^−5^, excluding all triallelic sites as well as reads with multiple best hits. A dendrogram was created using *hclust* v 3.6.2 and samples that were separated by a height of less than 0.15 were classified as clonal given that this height clustered replicate fragments of the same genet (Fig. S3). No clones were observed; however, one sample (AP4) was removed from further analyses due to its high divergence from all other samples in the dataset, which was strong evidence that it was an outlier (Fig. S3).

Further outlier examination was conducted using *arrayQualityMetrics* in *DESeq v1.39*^60^. One sample was flagged as an outlier and another was identified as having a high likelihood of being mislabeled (Fig. S4AC1). Both samples were removed from subsequent analyses (Table S4). To determine differentially expressed genes (DEGs), *DESeq2* v1.30^61^ was used with a correction for multiple testing done using the Benjamini and Hochberg method (FDR < 0.05)^62^. To test how symbiotic phenotype modulated the gene expression response, we conducted pairwise contrasts between either heat or cold challenge with control corals for each symbiotic phenotype separately. Lists of these DEGs isolated from each contrast were compared between symbiotic phenotypes using a Venn diagram. This process generated a list of unique and intersecting DEGs, which were used to perform a series of gene ontology (GO) enrichment analyses using Fisher’s exact tests within the GO_MWU R package^63^.

Global gene expression patterns were assessed by performing a variance stabilizing transformation (vst) followed by a principal component analysis (PCA). This PCA was then tested for differences between treatment levels using a permutational multivariate analysis of variance (PERMANOVA) with the *adonis* function in *vegan v2.5.4*^64^. In addition to the PCA, these findings were further evaluated by r-log transforming gene expression data followed by a discriminant function analysis (DAPC). Both the PCA and DAPC were given a gene expression plasticity score using a custom function^65^ based on distance between samples in the first two PC axes relative to the mean of all control samples. The effects of symbiotic phenotype and treatment on gene expression plasticity on both the PCA and DAPC were tested by first checking for assumptions of normality and equal variance followed by an ANOVA and Tukey’s honest significant differences post hoc tests.

### Colour analysis

Because *A. poculata* exists along a symbiosis continuum, we assessed the strength of our categorical symbiotic phenotype assignments, which were initially judged visually using brown or white phenotypes. Photos of each coral fragment were taken on the first, seventh, and last date of the experiment using a Coral Watch Coral Health reference card as a standard for light exposure^66^. All images were white balanced using Adobe Photoshop and then ten points on the coral were randomly selected using ImageJ. The red channel intensity was calculated from these points using a custom MATLAB script^67^ as a proxy for photobiont density. We then fit a linear mixed model, which included coral genotype as a random effect and symbiont phenotype, experimental day, and treatment as fixed effects.

### Evaluating the coral environmental stress response (ESR)

We first performed GO enrichment analysis using a Mann–Whitney U test (GO-MWU) based on rankings of signed log p-values^68^ for the heat and cold thermal challenges separately. In these analyses, we set parameters to filter GO categories if they contained more than 10% of the total number of genes, contain at least 10 genes to be considered, and the cluster cut height set to 0.01 to suppress merging of GO terms. This provides delta-ranks of each GO term, which quantifies the tendency of associated genes as being up- or downregulated in challenge samples vs controls. To evaluate the oxidative bleaching hypothesis, we explored signals of a response to ROS by isolating the children GO terms under the parent term *oxidative stress* (GO:0006979) using *GOfuncR v 1.10.0*^69^. This broadly captures GO terms associated with response to ROS and the average delta ranks were compared between treatments and symbiotic phenotypes using an ANOVA with fixed effects of treatment and symbiotic phenotype.

To characterise how the environmental stress response (ESR) differs across symbiotic phenotypes in each of the thermal challenges, we compared GO enrichment values from our data with results of a meta-analysis isolating the ESR in the genus *Acropora*^21^. While not a formal statistical analysis due to GO terms being non-independent from each other as they have overlapping gene sets, this presents a qualitative method to compare functional similarity across experiments. Here, a positive relationship would qualitatively indicate that the ESR is consistent with the Type “A” ESR and a negative relationship would suggest a Type “B” ESR. This comparison determines whether our thermal challenges elicit responses consistent with those observed in previous work conducted in tropical corals.

## Results

### Confirmation of symbiotic phenotype assignment

Analysis of coral colour showed that brown corals have significantly lower red channel intensity (*i.e.,* greater pigmentation) than white corals (beta = 54.96, 95% CI [43.09, 66.83], t(270) = 9.12, p < 0.001). There was also a negative interaction between day and heat challenge (*i.e.,* increasing pigmentation, beta =  − 0.88, 95% CI [− 1.51, − 0.25], t(270) =  − 2.75, p = 0.006). All other main effects and interactions between temperature, phenotype, and day were non-significant (refer to Table S2).

### Behavioural and gene expression responses of *Astrangia poculata* to thermal challenges

Both white and brown colonies reduced their polyp activity in response to food under cold (p < 0.001) and heat challenge (p = 0.012) relative to fragments under control conditions, and these responses were most pronounced when temperatures approached their extremes towards the end of the experiment (Fig. S1B). A significant interaction between symbiotic phenotype and heat challenge (p = 0.003) was also observed with brown fragments exhibiting less polyp activity than white fragments when temperatures increased. No interaction between symbiotic phenotype and cold challenge was observed (p = 0.083).

**Figure 1:**
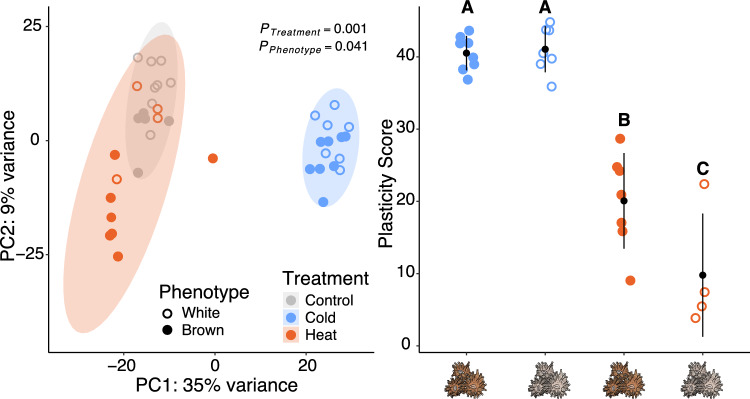
Gene expression responses to temperature challenges across symbiotic phenotypes in *Astrangia poculata*. (**A**) Principal component (PC) analysis of global expression of all *A. poculata* vst-normalized genes. Percentages represent the variance explained by each principal component (PC) and shaded areas represent 95% confidence ellipses within treatments. p-values indicate significant main effects of a factor using a permutational multivariate analysis of variance. (**B**) Mean gene expression plasticity of corals in thermal challenge treatments relative to control samples. Plasticity scores represent the distances of each coral fragment in a thermal challenge relative to the average expression of all control fragments across the first two PCs. Symbol and error bars are the modelled means and 95% confidence intervals. Letters depict significant differences in gene expression plasticity across treatments and symbiotic phenotypes based on Tukey’s honest significant differences post hoc test (see Table S3).

Both thermal challenges elicited strong transcriptome-wide changes in gene expression in *A. poculata* (Fig. 1A, *Adonis* p_treatment_ < 0.001). Corals in the cold challenge exhibited greater transcriptome plasticity (Fig. 1B, F(1, 22) = 143.35, p < 0.001, 95% CI [0.77, 1.00]) relative to those under heat challenge. This plasticity corresponded to approximately six times as many DEGs (FDR < 0.05) in cold challenge (6690 (19.9% of total genes) DEGs, 2549 (7.6%) upregulated, 4141 (12%) downregulated) compared to heat challenge (1011 (3%) DEGs; 552 (1.6%) upregulated, 459 (1.4%) downregulated).

### Response to heat challenge in *Astrangia poculata* is mediated by symbiotic phenotype

**Figure 2:**
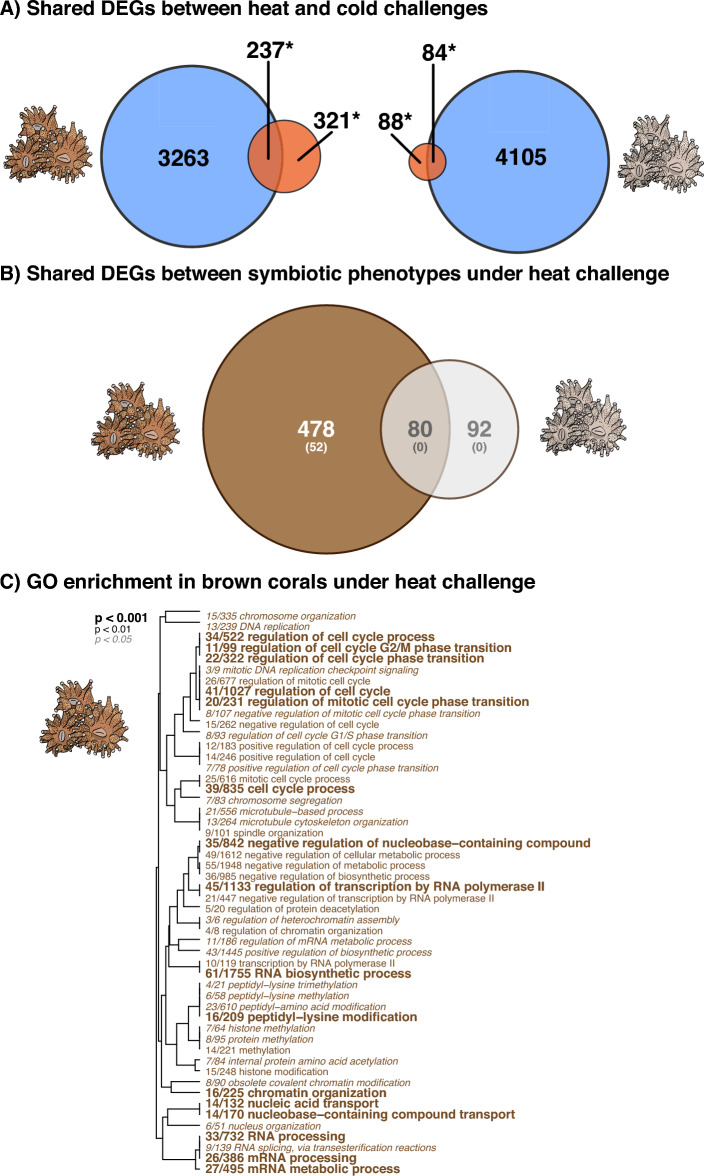
Functional responses of thermal-challenged *Astrangia poculata*. (**A**) Venn diagram of differentially expressed genes (DEGs, FDR < 0.05) from brown (left) and white (right) *A. poculata* fragments under heat challenge (red + asterisk) and cold challenge (blue) relative to control conditions. (**B**) Venn diagram of DEGs from the heat challenge (from asterisks above) between brown (brown) and white (grey) colonies. The top number denotes the number of DEGs and bottom number represents the number of enriched gene ontology (GO) terms. (**C**) GO enrichment results of the “biological processes (BP)” category derived from the list of unique DEGs that responded to heat challenge in the brown corals only. The dendrogram describes the relationship of shared genes between categories, and text size and boldness indicates the significance of each term.

Gene expression plasticity was significantly higher in brown corals compared to white corals under heat challenge (Tukey’s HSD, p = 0.0203; Fig. 1), and these patterns were confirmed by DAPC (Tukey’s HSD, p = 0.0047; Fig. S6). These differences in gene expression plasticity were consistent with the number of DEGs (Fig. 2), where brown corals had 558 (1.6%) DEGs (351 (1%) upregulated, 207 (0.62%) downregulated) under heat challenge compared to only 172 (0.5%) in heat-challenged white corals (61 (0.18%) upregulated, 111 (0.33%) downregulated).

A Venn diagram was constructed to highlight unique and shared lists of DEGs (Fig. 2). No GO enrichment was found within the list of unique DEGs from white corals under heat-challenge or the list of genes that were shared between brown and white corals under heat-challenge (intersection of venn diagram). In contrast, DEGs that were unique to brown corals under heat-challenge showcased significant GO enrichment across all GO hierarchies (FDR < 0.05; BP = 52, Fig. 2B; CC = 19, Fig. S9; MF = 13, Fig. S10) with two distinct clusters of related GO terms. The first cluster of terms was related to growth regulation (e.g., GO:0051726: regulation of cell cycle, GO:0022402: cell cycle process, GO:1901990: regulation of mitotic cell cycle phase transition) and the second the formation and metabolism of RNA (e.g., GO:0016071: mRNA metabolic process, GO:0032774: RNA biosynthetic process, GO:0018205: peptidyl-lysine modification, GO:0006357: regulation of transcription by RNA polymerase II, GO:0006325: chromatin organization).

### Characterising the environmental stress response in *Astrangia poculata*

**Figure 3:**
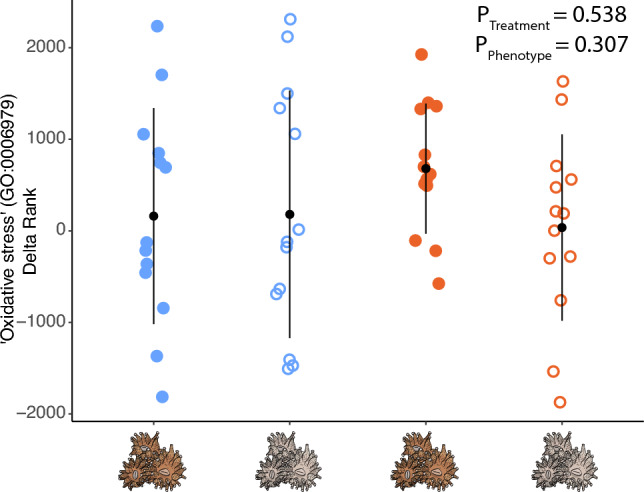
The oxidative stress response does not change between symbiotic phenotypes. Each dot represents the delta rank of one of the 13 children gene ontology (GO) terms under the oxidative stress parent term (GO:0006979) across thermal challenges within each symbiotic phenotype. Black dots represent mean delta rank values and error bars denote standard deviation.

To explore differential regulation of genes associated with oxidative stress, delta ranks of 13 children terms belonging to the parent GO term *oxidative stress* (GO:0006979) were explored. Delta-ranks of these children terms elicited similar responses under both heat and cold challenge (F(1, 48) = 0.38, p = 0.538), and this similarity was maintained across symbiotic phenotypes (Fig. 3; F(1, 48) = 1.07, p = 0.308), suggesting no change in the oxidative stress response across thermal challenges or symbiotic phenotypes.

**Figure 4:**
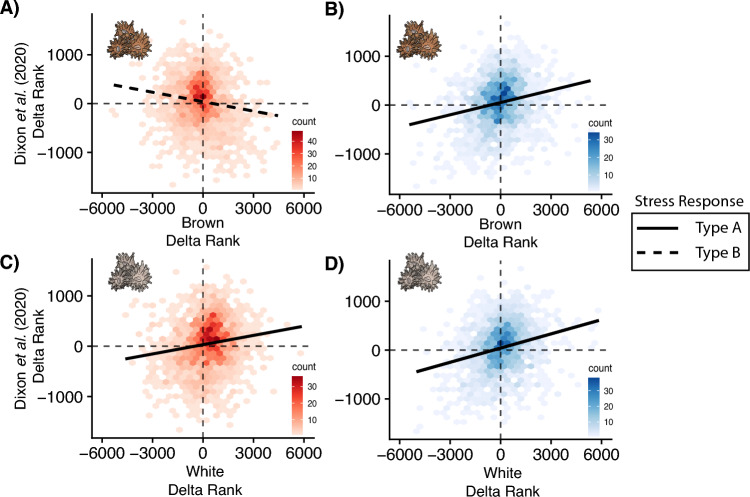
Environmental stress responses (ESR) of *Astrangia poculata*. Comparison of functional similarities in gene ontology (GO) delta ranks from studies that exhibited a Type A stress response (Dixon et al.^21^) compared with those from the heat (**A,C**) and cold challenge delta ranks (**B,D**) from white (**C,D**) and symbiotic states (**A,B**). Functional GO enrichment are shown for the biological processes (BP) and functional similarities between the two data sets would be characterised by a positive slope indicates that corals in a thermal challenge are eliciting a functionally similar response to that of tropical corals that have a severe ESR (Type ‘A’) whereas a negative slope suggests that the responses are more dissimilar therefore more similar to tropical corals exhibiting a moderate ESR (Type ‘B’).

To further explore the ESR beyond oxidative stress, delta ranks from Mann–Whitney U GO enrichment tests for each symbiotic phenotype and thermal challenge were contrasted with the delta ranks of the Type A stress response studies from Dixon et al*.*^21^. While not a formal statistic, a generally positive relationship suggests functional similarities between our data and that of the Type A stress studies in Dixon et al*.*^21^. There was a positive relationship for both symbiotic phenotypes under cold challenge in the ‘biological processes’ GO category (Fig. 4B,D), suggesting a Type A stress response. This positive slope was consistent in the ‘molecular function’ and ‘cellular component’ categories as well (Fig. S8B,C). In contrast, heat challenge elicited more dissimilar GO functionality as there was an opposing slopes for white and brown *A. poculata*, with white corals exhibiting a positive slope (Type A; Fig. 4C) across all GO categories while brown corals showcased a ‘Type B’ stress response as indicated by the negative slope (Fig. 4A [‘biological processes’ GO category], Fig. S9B,C [‘molecular function’ and ‘cellular component’ GO categories]).

## Discussion

Here, we conducted thermal challenge experiments on white and brown fragments of the facultative symbiotic coral *Astrangia poculata* with the broad goal of testing the influence of photosymbiosis on thermal stress responses. We predicted that under the oxidative bleaching hypothesis^25^ (Table 1), there would be an elevated environmental stress response (ESR; Type A response) due to the accumulation of photobiont and host derived reactive oxygen species (ROS) when thermally challenged. We explore responses of brown *A. poculata* in a state prior to exhibiting a bleaching phenotype under heat challenge and in fact find modest increases in pigmentation during the heat challenge for both symbiotic phenotypes (Fig. S2).

Overall, our results fail to support the oxidative bleaching hypothesis. Instead, we found that while photosymbiosis led to stronger gene expression plasticity in brown corals under heat challenge, this plasticity failed to showcase functional signatures consistent with the oxidative bleaching hypothesis. In contrast, our results highlight that coral gene expression patterns show enrichment for pathways involved in the regulation of symbiont growth via host cell cycle mechanisms under heat challenge^49,70^. This gene expression signature is consistent with the increased gene expression observed under heat challenge, and perhaps suggests that early in the heat stress response hosts have to dedicate energy to controlling symbiont proliferation^49^. In addition, our comparative analyses with a previous gene expression meta-analysis in tropical corals suggest that photosymbiosis is associated with a less strong ESR (Type B response) under heat challenge in this facultative coral system. However, under cold challenge, *A. poculata* exhibited stronger responses compared to heat challenge and photosymbiosis did not modulate these gene expression responses.

*A. poculata* experiences wide seasonal variation in temperature, including winter temperatures that are colder than the cold challenge applied here (Fig. S1C). Despite this, cold challenge elicited strong behavioural and transcriptomic responses in both white and brown colonies (Fig. 1, Fig. S7). While these results align with our previous findings in white *A. poculata*^22^, they also showcase that photosymbiosis failed to modulate this response. This pattern contrasts with a study in *A. poculata* that found that photosymbiosis mitigated host physiological responses under cold temperatures (8 °C), with brown colonies healing more quickly than white colonies^11^. It is possible that the colder temperature (4 °C) used in our study relative to Madin et al.^37^ elicited a stronger cold response, and this may have swamped out the effects of photosymbiosis. Nevertheless, the consistent strong gene expression plasticity observed under cold challenge is relevant given that cold water from arctic currents constrain *A. poculata* from expanding beyond Cape Cod^71^. Extreme cold weather therefore remains a salient stressor for *A. poculata* under climate change as Arctic warming influences upper-level atmospheric activity, which fuels severe winters^72^. These cold winters will therefore likely continue to constrain range expansion to higher latitudes in this species even as climate change progresses.

While photosymbiosis had little impact on host gene expression under cold challenge, previous studies have highlighted how photobiont biology is impacted by colder temperatures. The photobiont of *A. poculata* (*Breviolum psygmophilum*) exhibited reduced photochemical efficiency (F_v_/F_m_) as temperatures decreased, both *in hospite*^40^ and *ex hospite*^73^. While the *ex hospite* work initially showed a decrease in F_v_/F_m_, *B. psygmophilum* showed resilience and F_v_/F_m_ quickly recovered once temperatures returned to baseline^73^. While corals were not offered a recovery period in our study, this pattern in *A. poculata’s* photobiont suggests that hosts may also demonstrate similar resilience if they are co-adapted to their environments, which has been shown in other obligate symbiotic corals^74^. It has also been shown that *in hospite A. poculata* photobionts experience large seasonal variation in cell densities with lower photobiont densities observed in winter months^41^; however, this reduction in cell density can take several months of cold temperatures to manifest^75^. While cold challenge experiments are less common in coral reef studies given the imminent threat of ocean warming, previous work on photobiont physiology has showcased mixed results in tropical and subtropical coral species. In contrast to Sharp et al*.*^41^, a 10 week cold exposure (23 °C; control 26 °C) in *Acropora millepora* increased photobiont densities^13^, while a more severe cold challenge in tropical and subtropical *Porites lutea* populations (temperatures lowered from 26 to 12 °C over 18 days) reduced photobiont densities and photochemical efficiencies^76^. Clearly, the rate of cooling and severity of the temperature achieved likely dictate the magnitude and severity of photobiont responses and highlight the importance of these aspects in experimental studies^77^. It is also critical to point out that the rate of temperature change used here does not represent an ecologically relevant change for *A. poculata* given that these corals experience large differences in seasonal temperatures that occur over longer timescales.

Gene expression plasticity in response to cold challenge was notably large (Fig. 1), although no difference between symbiotic phenotypes was observed. A large response to a cold challenge was also observed in our previous work on white *A. poculata*^22^. Here we demonstrate that this strong gene expression response previously observed is consistent in brown corals, showcasing that photosymbiosis does not modulate response to cold challenge. We speculate that our sampling of *A. poculata* from their northern range edge may explain this plasticity. Plasticity can allow populations to establish in marginal habitats as they expand their ranges, so sampling along these range edges could be biased towards individuals with high plasticity^78,79^. Equally plausible, high gene expression plasticity may be explained by the climate variability hypothesis (CVH), which proposes that organisms experiencing high seasonal variability will exhibit higher plasticity facilitating acclimation across broad thermal regimes^80^. Unfortunately, our experiment is unable to discern the mechanisms underlying this high gene expression plasticity under cold challenge. Nevertheless, this pattern is consistent with previous work on high latitude, marginal populations of *Porites lutea,* which were found to not only exhibit higher cold tolerance, but also stronger transcriptomic responses when subjected to cold challenge relative to those sampled from the core tropical range^76^. Future comparative work on multiple populations of *A. poculata* is warranted.

Relative to cold challenge, we observed muted gene expression responses (Fig. 1) to heat challenge despite experimental temperatures exceeding temperatures experienced at the collection site (Fig. S1). A large plastic response to cold, but not heat challenge is seemingly at odds with both the marginal environment hypothesis and the CVH, as both would predict highly plastic responses to both temperature treatments. However, it is possible that short term cold challenges are more stressful for *A. poculata* than short term heat challenges. For example, experimental work on the tropical coral *Acropora yongei* found that short term cold stress was more detrimental than short term heat stress, but longer-term elevated temperatures were ultimately more harmful than longer-term cold temperatures^81^. Alternatively, the rate of cooling may have a stronger influence than the rate of warming on *A. poculata.* Given that this *A. poculata* population is known to enter a hibernation-like state termed quiescence after long seasonal decreases in temperature^82^, perhaps more gradual temperature decreases would induce acclimatory responses that lead to quiescence rather than the strong ESR observed here. In addition, these colonies were acclimated to ambient room temperature, which is relatively warmer than the average temperature experienced from the collection site. This acclimation may have impacted their response to the heat challenge, and future experimental work comparing responses in winter- *versus* summer-acclimated colonies would be exciting. Lastly, we posit that this muted response to heat challenge may be further evidence that Woods Hole, MA represents a marginal habitat for *A. poculata* and that these corals are adapted to more subtropical locations (e.g. Virginia). Indeed, high gene flow between southern (i.e., North Carolina) and northern (i.e., Massachusetts) populations has been documented^83^, highlighting a need for future reciprocal transplant experiments between these locations to test this hypothesis.

In response to heat challenge, brown *A. poculata* exhibited higher gene expression plasticity with three times as many DEGs and a reduced feeding response compared to white *A. poculata*. This reduced feeding may indicate stress in brown corals, however, white corals may need to maintain higher levels of heterotrophy due to more limited supplemental nutrition. In addition, our GO analysis highlighted genes involved in cell cycle processes and not response to stress as predicted (Fig. 2). This pattern of higher gene expression plasticity under heat challenge in brown corals perhaps makes sense given their increased need to maintain homeostasis across additional cell types (i.e., symbiosomes) when compared to their white counterparts^3^. Interestingly, we did not observe gene expression signatures consistent with increased respiration, which has been previously shown in corals experiencing extreme acidification and was linked with increased energy expenditure associated with maintaining homeostasis^84^. This interaction between photobionts and hosts leading to changes in cell cycle processes is relevant as the division of host and photobiont cells are often synchronized^85^, preventing photobiont overpopulation, which can lead to the mutualism shifting to parasitism^86^. Furthermore, one mechanism that hosts use to modulate photobiont growth is by maintaining the space available for photobiont cells to grow into (for review, see^87^). Similar GO enrichment between brown and white tissues have been observed in the facultatively symbiotic coral *Oculina arbuscula* under baseline conditions^49^, further showcasing that controlling symbiont cell densities is a critical aspect of symbiosis maintenance. Interestingly, research has shown that cell densities can increase under short term thermal challenge, which can lead to higher symbiont loads that may increase ROS production within hosts^88^. Because we did not observe bleaching and instead observed increases in pigmentation, it is possible that the signature of increased cell cycle control in the gene expression of brown corals under heat stress serves as a precursor to bleaching itself. Perhaps, the first sign of dysbiosis in brown corals is the increased regulation of photobiont replication under rising temperatures, which may serve to limit oxidative stress to the host. Under this scenario it is possible that prolonged heat challenge would lead to the host losing control over photobiont growth, which would lead to the buildup of ROS and the ultimate loss of photobionts (i.e., bleaching). This contrasts with recent work suggesting that corals can farm and digest excess symbiont cells^89^, which would simultaneously increase nutrient availability to the host and control cell densities. Overall, we posit that under heat challenge, *A. poculata* exhibits enrichment of cell cycle processes, which are necessary to control symbiont cell densities under warmer temperatures. Given the limited duration of our experiment, further work examining intracellular ROS production coupled with physiological traits across a variety of temperature challenges and durations is warranted.

At first, we hypothesised that the observed elevated gene expression plasticity under heat challenge in brown corals would be accompanied by a greater ESR, which would be driven by accumulation of photobiont derived ROS (Table 1). However, this higher gene expression plasticity was notably driven by variation along the second principal component axis whereas cold challenge explained variation along the first principal component (Fig. 1). Together, this pattern suggests that the interaction between photosymbiosis and heat challenge impact different mechanistic pathways from cold challenge. This pattern is corroborated by our exploration of genes belonging to GO terms nested within “oxidative stress”, which demonstrated no differences in the enrichment of these terms between white and brown colonies under either thermal challenge (Fig. 3). Therefore, this pattern does not support the hypothesis of increased ROS produced from algal photobionts in brown corals under heat challenge and necessitates further work measuring ROS directly to confirm our findings.

This pattern of symbiosis lessening the ESR could be due to constitutively higher antioxidant and stress mitigation mechanisms when corals are in photosymbiosis, perhaps mediated through energetic increases associated with symbiont digestion^89^. For example, melanin and catalase levels, which play roles in mitigating stress, have been found to be higher in brown *A. poculata* relative to white colonies^43^. While it is possible that our sampling for gene expression prior to bleaching led to our inability to capture a signal of increased oxidative stress, we speculate that this pattern supports the growing evidence that ROS accumulation from photoinhibition may not be the first step in the coral bleaching process^90^. For example, ROS accumulation in *Aiptasia* after a heat shock was determined to be host derived and generated prior to photobiont photoinhibition^31^. In addition, when *Aiptasia* anemones were treated with the exogenous antioxidant mannitol, there was a significant increase in photobiont-derived ROS under elevated temperatures^91^. Despite this increased photobiont-derived ROS, the treatment mitigated bleaching, suggesting that ROS from the photobiont alone is insufficient to cause bleaching. Furthermore, coral bleaching can occur under complete darkness, suggesting that this process can be independent of photosynthesis alltogether^92^. These results have reshaped our understanding of the early onset of bleaching, shifting the focus from photo-induced ROS accumulation towards the recognition of nutritional mechanisms potentially altering responses to thermal stress^33^. However, it is important to acknowledge that temperate corals may exhibit inherently different physiology than tropical corals as they are less reliant on photosynthetically derived sources of energy. In addition, corals hosting different Symbiodiniaceae genera have been shown to exhibit different physiology^93^, gene expression^94^ and ultimately exhibit different bleaching responses^95^. As *A. poculata* are known to exclusively host *Breviolum psygmophilum*^73^, which potentially limits its use as a model system for bleaching.

Lastly, to further explore the ESR beyond oxidative stress, we qualitatively compared our findings with a meta-analysis of coral gene expression responses to stress^21^. While not a formal statistical analysis and caution is warranted in over interpreting these results, this approach can be used to broadly assess the type of ESRs elicited by the thermal challenges between symbiotic phenotypes^22,23^. Interestingly, we observed divergent ESRs to heat challenge between symbiotic phenotypes, with expression patterns of white corals consistent with a ‘Type A’ response (positive slope) and brown corals a ‘Type B’ response (negative slope). Type B responses are typically observed under moderate stress^21^, suggesting that brown *A. poculata* exhibited a less severe ESR than white corals under heat challenge. This result contrasts previous work demonstrating that white *A. poculata* exhibited a Type B response to heat challenges^22^, and higher thermal optima compared to brown corals^48^. However, these results are challenging to contextualize without a full understanding of where these thermal challenge treatments fall relative to the thermal maximum of the sampled population. Future work exploring whether thermal challenges beyond a population’s critical thermal maximum shift this ESR under heat challenge is warranted.

Ultimately, the mechanisms underlying how photosymbiosis reduces the ESR under heat challenge remain to be determined. Photobionts may lessen stress by protecting the coral from the compounding effects of light stress. As photobionts themselves are pigmented, they block or absorb light that would otherwise be scattered and amplified by the coral skeleton^96,97^, potentially limiting the additive effects of temperature and light stress. Alternatively, photobionts provide carbon sugars to the host, so this additional energy input may mitigate ESRs in brown corals under heat challenge^35^. Furthermore, hosts may farm and consume these algae for excess nutrition^89^. Future work aiming to disentangle how photosymbiosis mitigates the ESR in brown *A. poculata* would benefit from a deeper understanding of (1) *A. poculata*’s thermal maximum, (2) how the rate of temperature increases impacts physiology, (3) nutrient exchange mechanisms in this facultative symbiosis (but see^4,42^) how changes in symbiont density shift these responses. Overall, these findings showcase the potential complexities of photosymbiosis in this system and fail to implicate the symbiont in amplifying the coral ESR under heat.

### Supplementary Information

Supplementary Information.

## Data Availability

Raw sequences are made available from the NCBI SRA under accession PRJNA1013245. Full reproducible data and code as well as all intermediate files are available at https://github.com/wuitchik.
