# Supplementary material for: Photosymbiosis reduces the environmental stress response under a heat challenge in a facultatively symbiotic coral
Source: Sci Rep. 2024 Jul 5;14:15484. doi: 10.1038/s41598-024-66057-2 (PMC11226616; doi:10.1038/s41598-024-66057-2)
Supplement: Supplementary file 1 — Supplementary Information. [file 41598_2024_66057_MOESM1_ESM.docx]

**Supplemental Information**

*
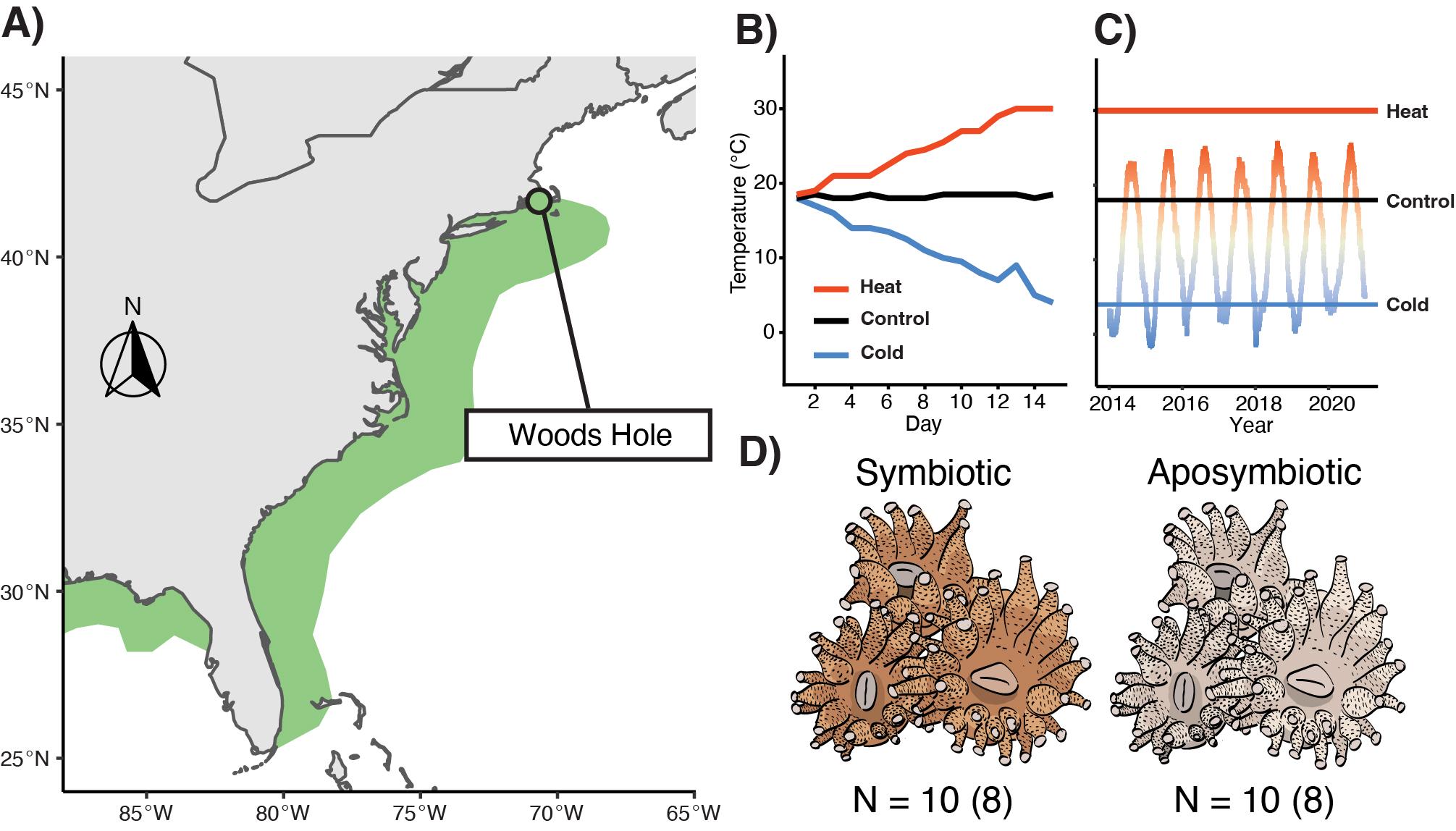
*

Figure S1 |*Astrangia poculata* thermal challenge experimental design. A) Map of the eastern seaboard of the United States with documented *Astrangia poculata* distributions in green (distributions based on Thornhill et al., 2008). B) Temperature data for three experimental treatments throughout a 15 day thermal challenge. C) Mean hourly temperature profiles at Woods Hole, MA with final temperature reached in each challenge treatment overlayed (Black: Control, Red: Heat, Blue: Cold). Colour gradient shifts from red (maximum temperature) to grey (mean temperature) to blue (minimum temperature). Seasonal temperature data were obtained from the National Oceanic and Atmospheric Administration (NOAA) weather buoy number BZBM3. D) Symbiosis phenotypes of *A. poculata* with sample sizes used for behavioural assay (gene expression analyses in parentheses).


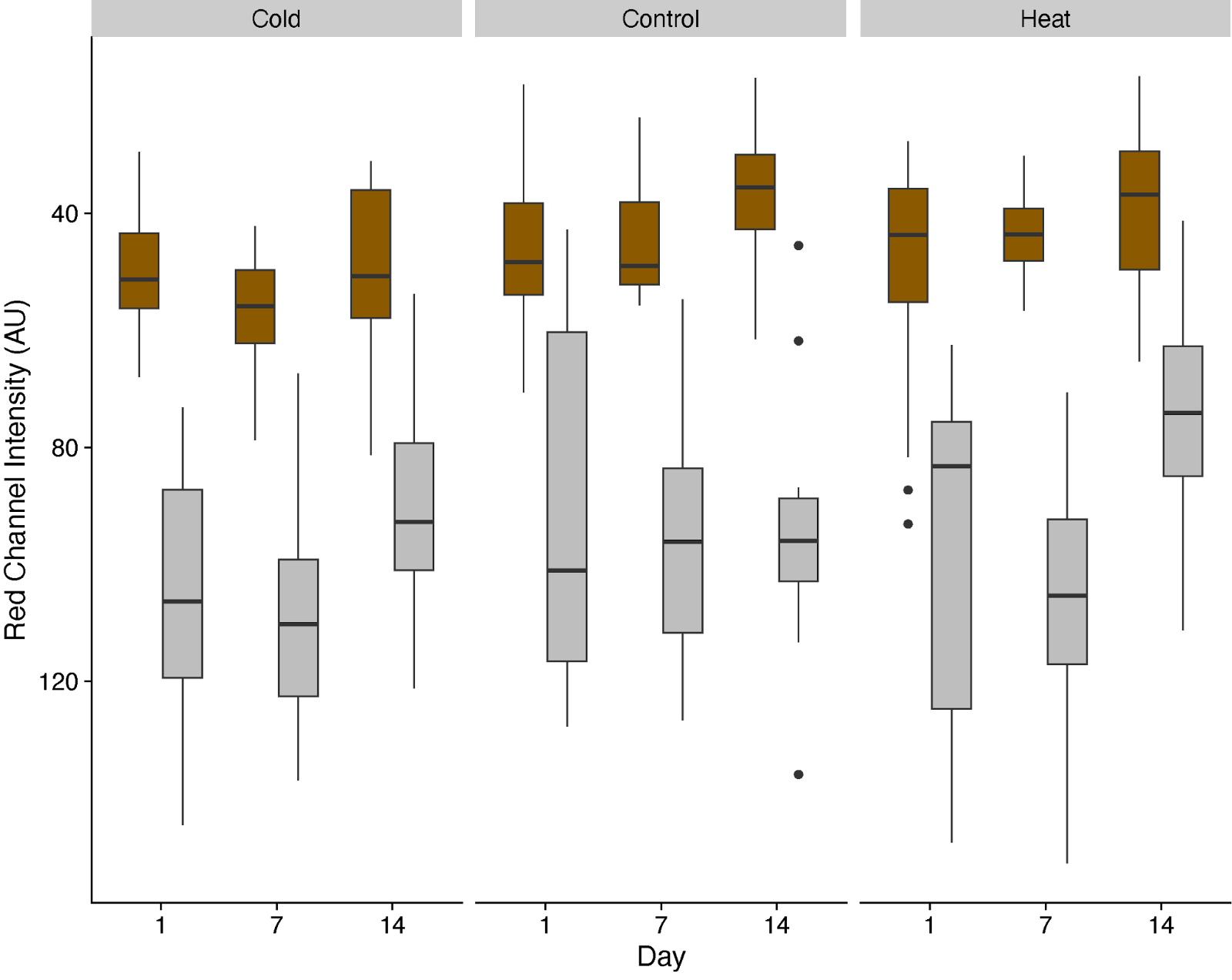


Figure S2 | Mean intensity of the red channel from photograph analysis in arbitrary units where increased values denote lower tissue colour across temperatures and symbiotic phenotypes.The y-axis has been inverted to showcase that lower red intensity values have greater colouration. Refer to Table S1 for a summary of significant effects using a linear mixed effects model treating temperature, day and phenotype as fixed effects and genotype as a random effect.

##### Table S1 | Sample size post filtering and outlier removal used for gene expression analyses

​​

|  | Heat Challenge | Control | Cold Challenge |
| --- | --- | --- | --- |
| Symbiotic | 7 | 5 | 8 |
| Aposymbiotic | 4 | 8 | 7 |

Table S2 | Summary of fixed effects from the linear mixed effect model of red channel intensity from photographic analyses of brown and white symbiotic phenotypes across experimental day.

|  | Estimate | Std. Error | df | t value | Pr(>\|t\|) |
| --- | --- | --- | --- | --- | --- |
| (Intercept) | 43.574 | 4.06 | 133.693 | 10.741 | 8.72E-20 |
| Phenotype | 54.962 | 6.03 | 109.951 | 9.118 | 4.12E-15 |
| Day | -0.191 | 0.25 | 183.481 | -0.780 | 0.436 |
| Cold | 10.550 | 5.57 | 125.966 | 1.894 | 0.060 |
| Heat | 8.383 | 5.64 | 126.036 | 1.486 | 0.140 |
| Phenotype:Day | -0.436 | 0.27 | 183.965 | -1.647 | 0.101 |
| Day:Cold | -0.074 | 0.31 | 183.365 | -0.237 | 0.813 |
| Day:Heat | -0.880 | 0.32 | 184.431 | -2.750 | 0.007 |
| White:Cold | -2.224 | 8.13 | 88.898 | -0.274 | 0.785 |
| White:Heat | -3.623 | 8.18 | 89.537 | -0.443 | 0.659 |


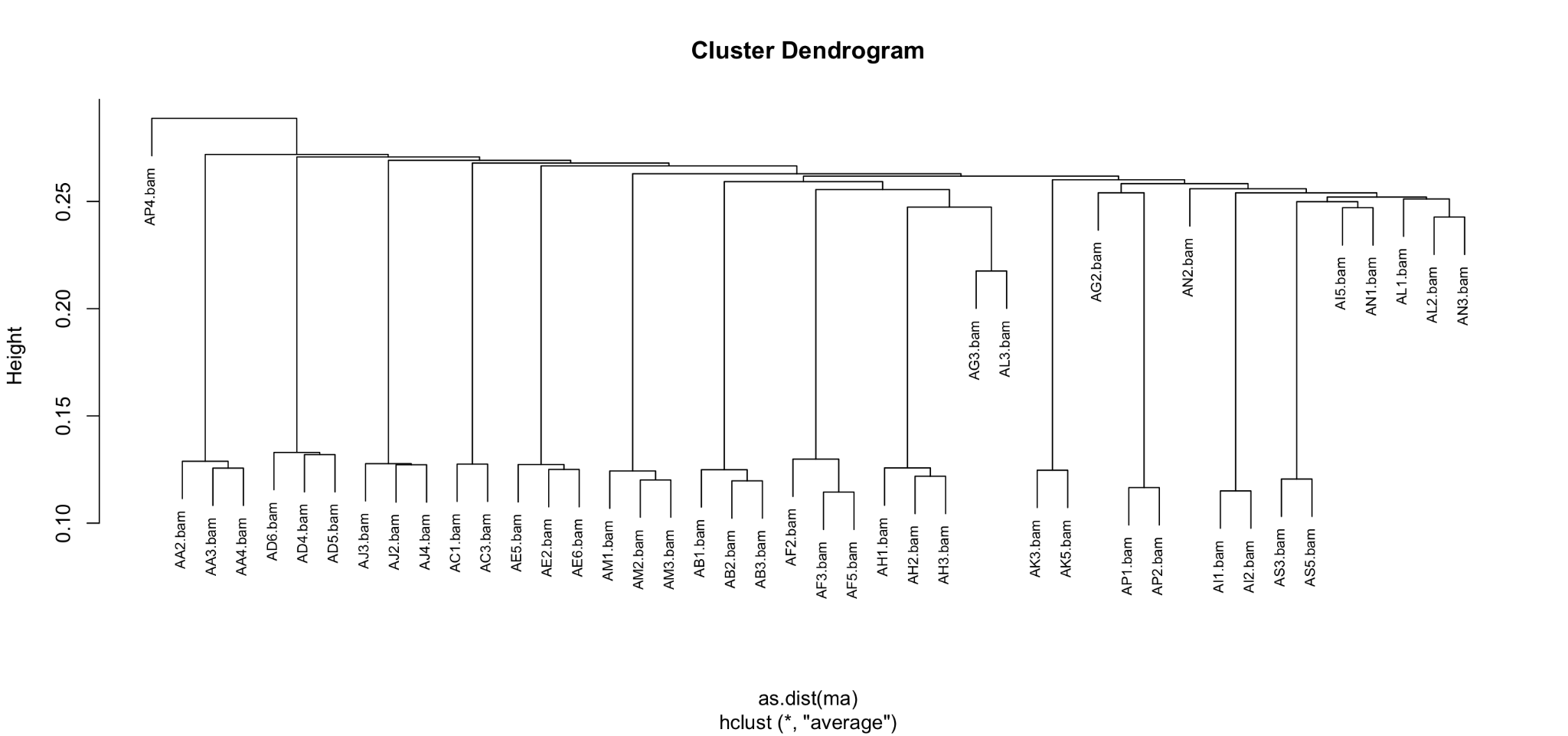


##### Figure S3| Cluster dendrogram of *Astrangia poculata* to identify clonal individuals from SNPs. Sample AP4 was removed from analyses due to its high divergence between all other samples.


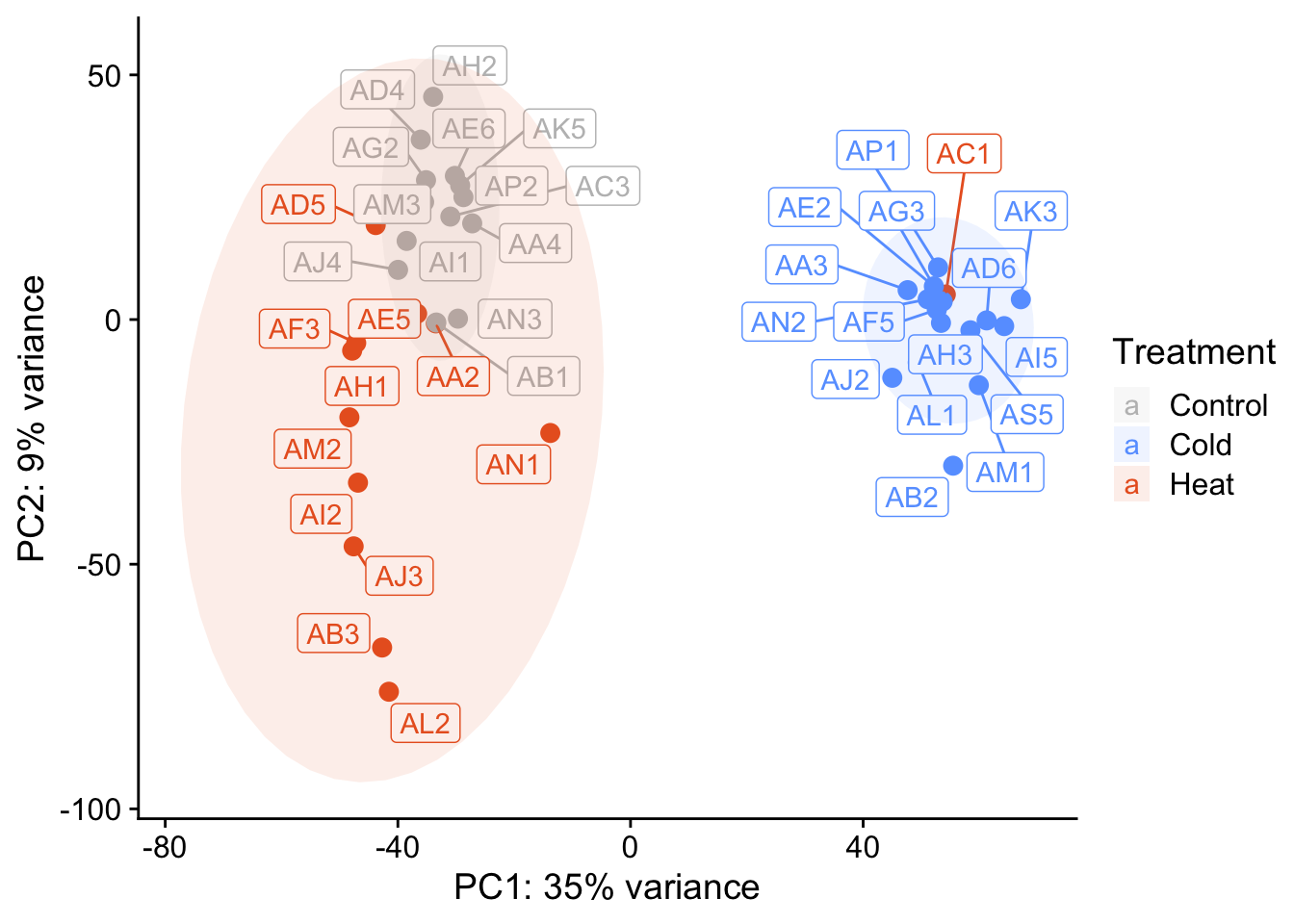


##### Figure S4. Gene expression responses to temperature challenge treatments prior to sample “AC1” removal. Principal component analysis of overall expression of all *A. poculata* vst-normalized genes. Percentages represent the variance explained by each axis and shaded areas represent 95% confidence ellipses within treatments. Sample AC1 was removed due to its high divergence in gene expression profile.

**
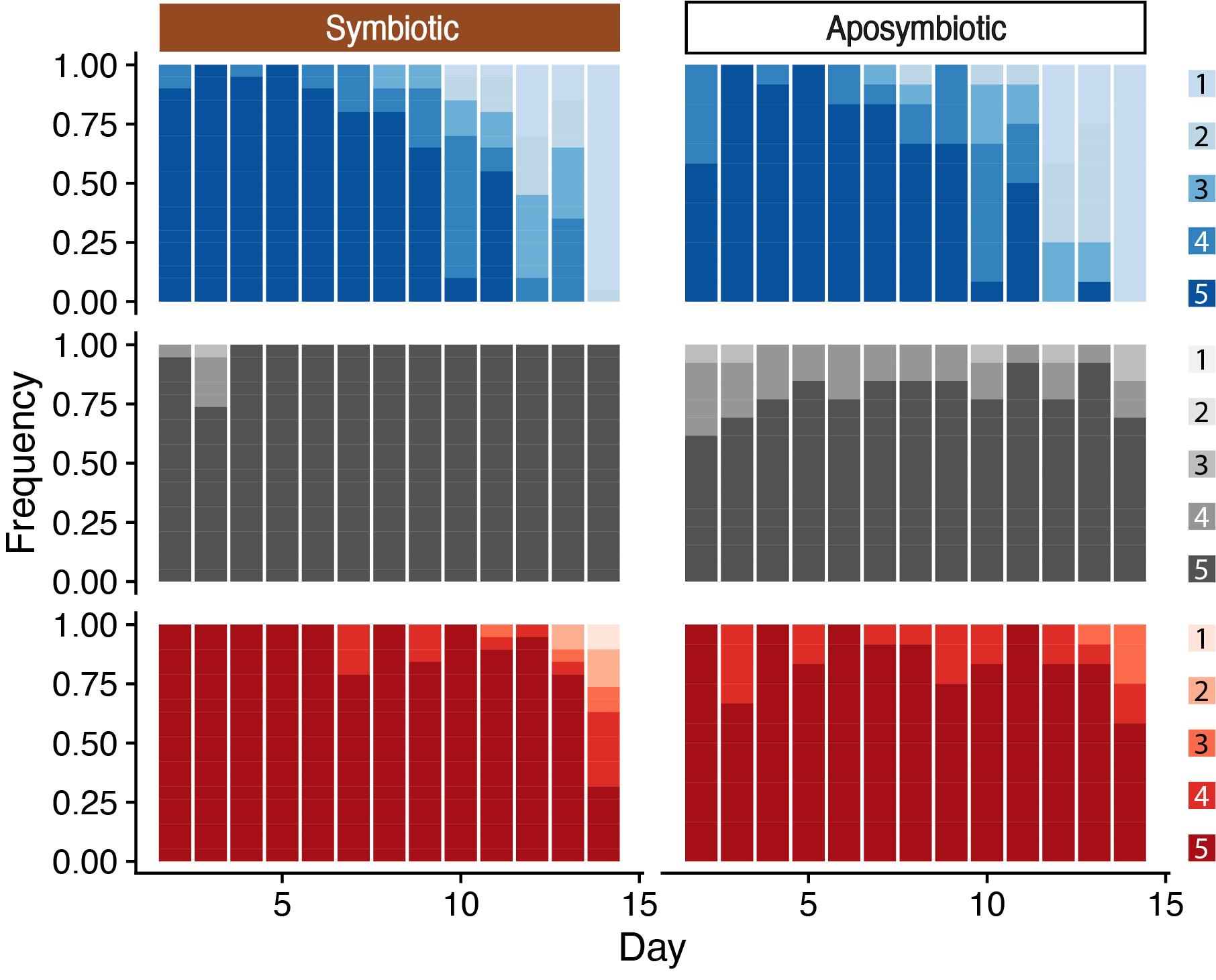
**

##### **Figure S5.** Behavioural response to food stimuli. Polyp activity was measured as the proportion of polyps that were extended per coral fragment (approximately, 1 = 0%, 2 = 25%, 3 = 50%, 4 = 75%, 5 = 100%) across the 15 day experiment. Cold challenge (Blues; top), control (Greys; middle) and heat challenge (Reds; bottom) with respective legend showing behavioural scores.


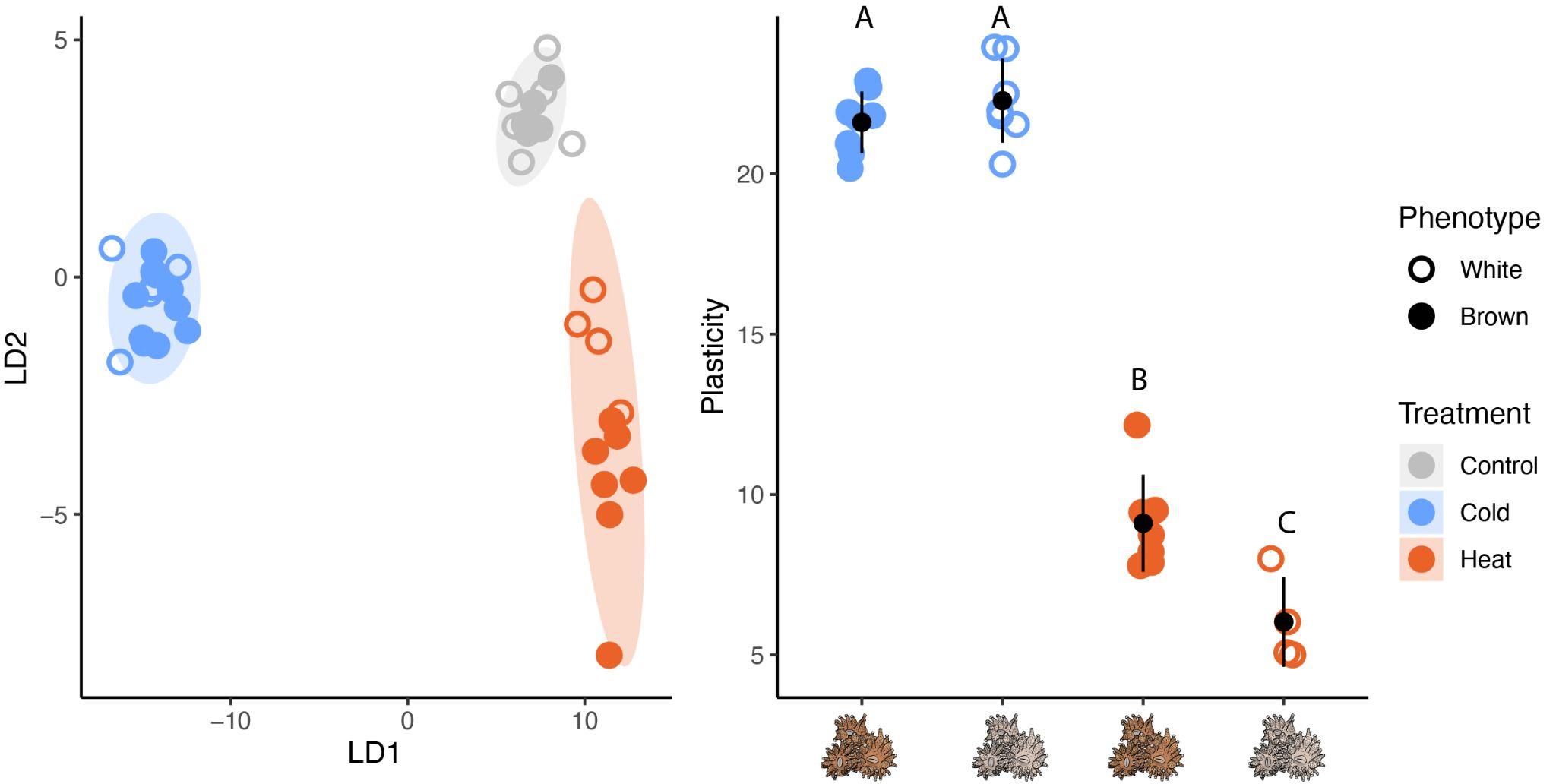


##### Figure S6. A) Discriminant function analysis of overall expression of all *A. poculata* rlogged-normalized genes. Shaded areas represent 95% confidence ellipses within treatments. B) Mean gene expression plasticity of corals in thermal challenge treatments relative to control samples. Plasticity scores represent the first two principal component distances of each coral fragment in a thermal challenge treatment relative to the average expression of all control fragments. Symbol and error bars are the modeled means and 95% confidence interval. Letters depict significant differences in gene expression plasticity across treatments and symbiotic states based on Tukey’s honest significant differences post hoc test.

Table S3. Summary of Tukey’s honest significant difference post hoc test following an ANOVA using treatment and phenotype as fixed effects on gene expression plasticity.

|  | diff | lwr | upr | padj |
| --- | --- | --- | --- | --- |
| Cold_White-Cold_Brown | 0.537 | -6.853 | 7.926 | 0.997 |
| Heat_Brown-Cold_Brown | -20.448 | -27.838 | -13.058 | 6.55E-07 |
| Heat_White-Cold_Brown | -30.732 | -39.476 | -21.989 | 1.08E-08 |
| Heat_Brown-Cold_White | -20.985 | -28.617 | -13.353 | 7.26E-07 |
| Heat_White-Cold_White | -31.269 | -40.218 | -22.320 | 1.21E-08 |
| Heat_White-Heat_Brown | -10.284 | -19.233 | -1.335 | 0.0204 |

##### Table S4 | Summary of behavioural ordinal logistic regression treating genotype and system as random effects.

|  | **Estimate** | **Std. Error** | **z value** | **Pr(>\|z\|)** |
| --- | --- | --- | --- | --- |
| Cold Challenge | 4.886 | 0.835 | 5.854 | **<0.001** |
| Heat Challenge | 1.960 | 0.783 | 2.503 | **0.012** |
| Symbiotic State | -1.035 | 1.228 | -0.844 | 0.399 |
| Experimental day Day | 0.059 | 0.053 | 1.116 | 0.264 |
| Cold Challenge * Symbiotic State | 2.653 | 1.531 | 1.733 | 0.083 |
| Heat Challenge * Symbiotic State | 5.460 | 1.815 | 3.009 | **0.003** |
| Cold Challenge * Experimental Day | -0.771 | 0.089 | -8.624 | **<0.001** |
| Heat Challenge * Experimental Day | -0.188 | 0.085 | -2.212 | **0.027** |
| Symbiotic State * Day | 0.782 | 0.354 | 2.208 | **0.027** |
| Cold Challenge * Symbiotic state * Experimental Day | -0.904 | 0.366 | -2.470 | **0.014** |
| Heat Challenge * Symbiotic State * Experimental Day | -1.179 | 0.375 | -3.146 | **0.002** |

##### **Table S4.** Read counts and sample notes at various steps of the mapping process.

| Sample | Genotype | Treatment | Phenotype | Raw total reads | Trimmed total  reads | Mapped reads to host | Percent  mapped to host | Photobiont counts | Relative  Photobiont Counts | Notes |
| --- | --- | --- | --- | --- | --- | --- | --- | --- | --- | --- |
| AA2 | A | Heat | White | 5057078 | 1389360 | 970628 | 70% | 5929 | 0.60% |  |
| AA3 | A | Cold | White | 3740666 | 876102 | 574992 | 66% | 2309 | 0.40% |  |
| AA4 | A | Control | White | 2780124 | 837632 | 595368 | 71% | 11632 | 2.00% |  |
| AB1 | B | Control | Brown | 5130310 | 1399027 | 1010691 | 72% | 30109 | 3.00% |  |
| AB2 | B | Cold | Brown | 5607990 | 1568156 | 585488 | 37% | 23201 | 4.00% |  |
| AB3 | B | Heat | Brown | 810976 | 301017 | 226910 | 75% | 10972 | 4.80% |  |
| AC1 | C | Heat | White | 6186284 | 1808120 | 1245649 | 69% | 3396 | 0.30% | Removed based on PCA |
| AC3 | C | Control | White | 8772244 | 2254587 | 1535196 | 68% | 6770 | 0.40% |  |
| AD4 | D | Control | White | 5621719 | 1178831 | 834049 | 71% | 1264 | 0.20% |  |
| AD5 | D | Heat | White | 2609448 | 702086 | 269066 | 38% | 7010 | 2.60% |  |
| AD6 | D | Cold | White | 6092666 | 1545354 | 1170859 | 76% | 6111 | 0.50% |  |
| AE2 | E | Cold | White | 3056318 | 1042104 | 777881 | 75% | 1042 | 0.10% |  |
| AE5 | E | Heat | White | 4528236 | 1193039 | 546344 | 46% | 4775 | 0.90% |  |
| AE6 | E | Control | White | 5024737 | 1294243 | 762508 | 59% | 3929 | 0.50% |  |
| AF2 | F | Control | Brown | 8447 | 4222 | 3509 | 83% | 46 | 1.30% | Removed due to low counts and because of identified outlier on arrayQualitymetrics |
| AF3 | F | Heat | Brown | 3844256 | 949292 | 546121 | 58% | 2442 | 0.40% |  |
| AF5 | F | Cold | Brown | 5017655 | 1394088 | 976195 | 70% | 24009 | 2.50% |  |
| AG1 | G | Heat | White | 51 | 28 | 21 | 75% | 0 | 0.00% | Removed due to low counts |
| AG2 | G | Control | White | 5632433 | 1586345 | 1141961 | 72% | 41035 | 3.60% |  |
| AG3 | G | Cold | White | 6128832 | 1645995 | 1257027 | 76% | 15370 | 1.20% |  |
| AH1 | H | Heat | White | 4562147 | 940299 | 665266 | 71% | 3100 | 0.50% |  |
| AH2 | H | Control | White | 2653474 | 454304 | 340981 | 75% | 1883 | 0.60% |  |
| AH3 | H | Cold | White | 2471788 | 790449 | 609247 | 77% | 8044 | 1.30% |  |
| AI1 | I | Control | Brown | 4720194 | 1334265 | 1009054 | 76% | 11998 | 1.20% |  |
| AI2 | I | Heat | Brown | 1101842 | 431060 | 318267 | 74% | 12738 | 4.00% |  |
| AI5 | I | Cold | Brown | 1655390 | 630365 | 487477 | 77% | 8760 | 1.80% |  |
| AJ2 | J | Cold | Brown | 2983572 | 955180 | 689278 | 72% | 9467 | 1.40% |  |
| AJ3 | J | Heat | Brown | 2565084 | 775431 | 557771 | 72% | 23536 | 4.20% |  |
| AJ4 | J | Control | Brown | 4827011 | 940906 | 613723 | 65% | 5264 | 0.90% |  |
| AK2 | K | Heat | White | 107 | 70 | 38 | 54% | 1 | 2.60% | Removed due to low counts |
| AK3 | K | Cold | White | 3009774 | 690136 | 511510 | 74% | 1143 | 0.20% |  |
| AK5 | K | Control | White | 3280922 | 1012242 | 768666 | 76% | 4163 | 0.50% |  |
| AL1 | L | Cold | Brown | 3298436 | 979125 | 665532 | 68% | 17685 | 2.70% |  |
| AL2 | L | Heat | Brown | 4785674 | 569249 | 434299 | 76% | 10598 | 2.40% |  |
| AL3 | L | Control | Brown | 27109 | 10644 | 8522 | 80% | 240 | 2.80% | Removed due to low counts |
| AM1 | M | Cold | Brown | 2797047 | 609399 | 374660 | 61% | 6682 | 1.80% |  |
| AM2 | M | Heat | Brown | 3510007 | 1041855 | 748704 | 72% | 10108 | 1.40% |  |
| AM3 | M | Control | Brown | 5028286 | 1193612 | 866928 | 73% | 3290 | 0.40% |  |
| AN1 | N | Heat | Brown | 3444755 | 1130026 | 851746 | 75% | 19778 | 2.30% |  |
| AN2 | N | Cold | Brown | 4278155 | 1368617 | 1020334 | 75% | 40635 | 4.00% |  |
| AN3 | N | Control | Brown | 3587592 | 847269 | 555903 | 66% | 16331 | 2.90% |  |
| AP1 | P | Cold | White | 4284600 | 1339960 | 882049 | 66% | 2883 | 0.30% |  |
| AP2 | P | Control | White | 1997983 | 626374 | 482611 | 77% | 1937 | 0.40% |  |
| AP4 | P | Heat | White | 2456638 | 805254 | 569111 | 71% | 4428 | 0.80% | Removed due to hclust outlier |
| AS1 | S | Control | Brown | 37 | 24 | 16 | 67% | 0 | 0.00% | Removed due to low counts |
| AS3 | S | Heat | Brown | 4066912 | 417483 | 291188 | 70% | 29108 | 10.00% | Identified as outlier in arrayQuality Metrics |
| AS5 | S | Cold | Brown | 2041873 | 668257 | 475513 | 71% | 9555 | 2.00% |  |
|  |  |  |  |  |  |  |  |  |  |  |
|  |  |  | average | 3512487 | 926202 | 634657 | 69% | 9887 | 1.80% |  |
|  |  |  | min | 37 | 24 | 16 | 37% | 0 | 0.00% |  |
|  |  |  | max | 8772244 | 2254587 | 1535196 | 83% | 41035 | 10.00% |  |

##### Table S4. Summary of behavioural ordinal logistic regression treating genotype and system as random effects.

|  | **Estimate** | **Std. Error** | **z value** | **Pr(>\|z\|)** |
| --- | --- | --- | --- | --- |
| Cold Challenge | 4.886 | 0.835 | 5.854 | **<0.001** |
| Heat Challenge | 1.960 | 0.783 | 2.503 | **0.012** |
| Symbiotic State | -1.035 | 1.228 | -0.844 | 0.399 |
| Experimental day Day | 0.059 | 0.053 | 1.116 | 0.264 |
| Cold Challenge * Symbiotic State | 2.653 | 1.531 | 1.733 | 0.083 |
| Heat Challenge * Symbiotic State | 5.460 | 1.815 | 3.009 | **0.003** |
| Cold Challenge * Experimental Day | -0.771 | 0.089 | -8.624 | **<0.001** |
| Heat Challenge * Experimental Day | -0.188 | 0.085 | -2.212 | **0.027** |
| Symbiotic State * Day | 0.782 | 0.354 | 2.208 | **0.027** |
| Cold Challenge * Symbiotic state * Experimental Day | -0.904 | 0.366 | -2.470 | **0.014** |
| Heat Challenge * Symbiotic State * Experimental Day | -1.179 | 0.375 | -3.146 | **0.002** |


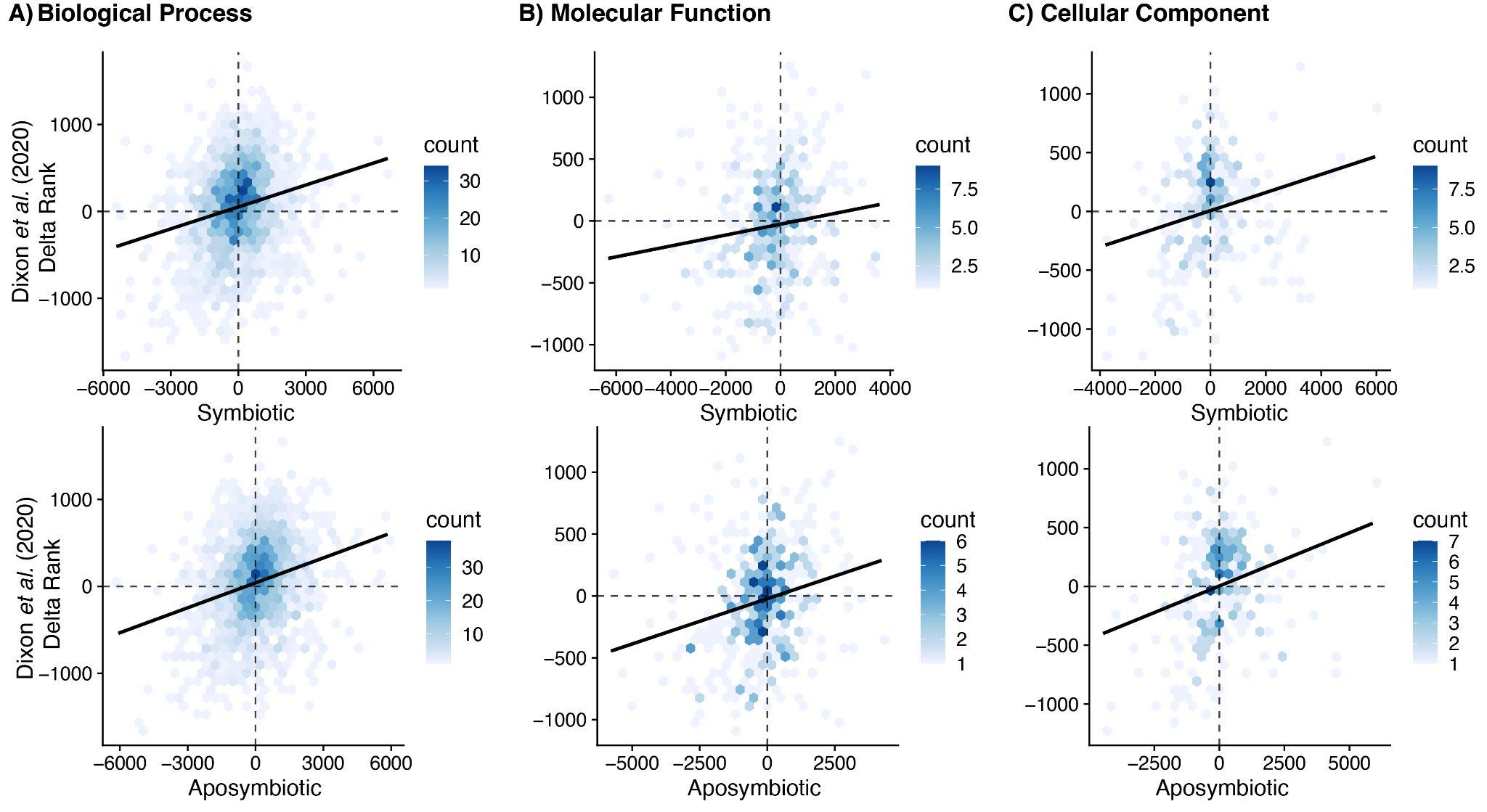


##### **Figure S7 |** Comparison of gene ontology (GO) delta ranks from environmental stress response studies featured in (G. Dixon et al., 2020) with cold challenge. A positive slope indicates that a coral is showing signatures of severe environmental stress response (ESR) whereas a negative slope is more characteristic of moderate ESR.


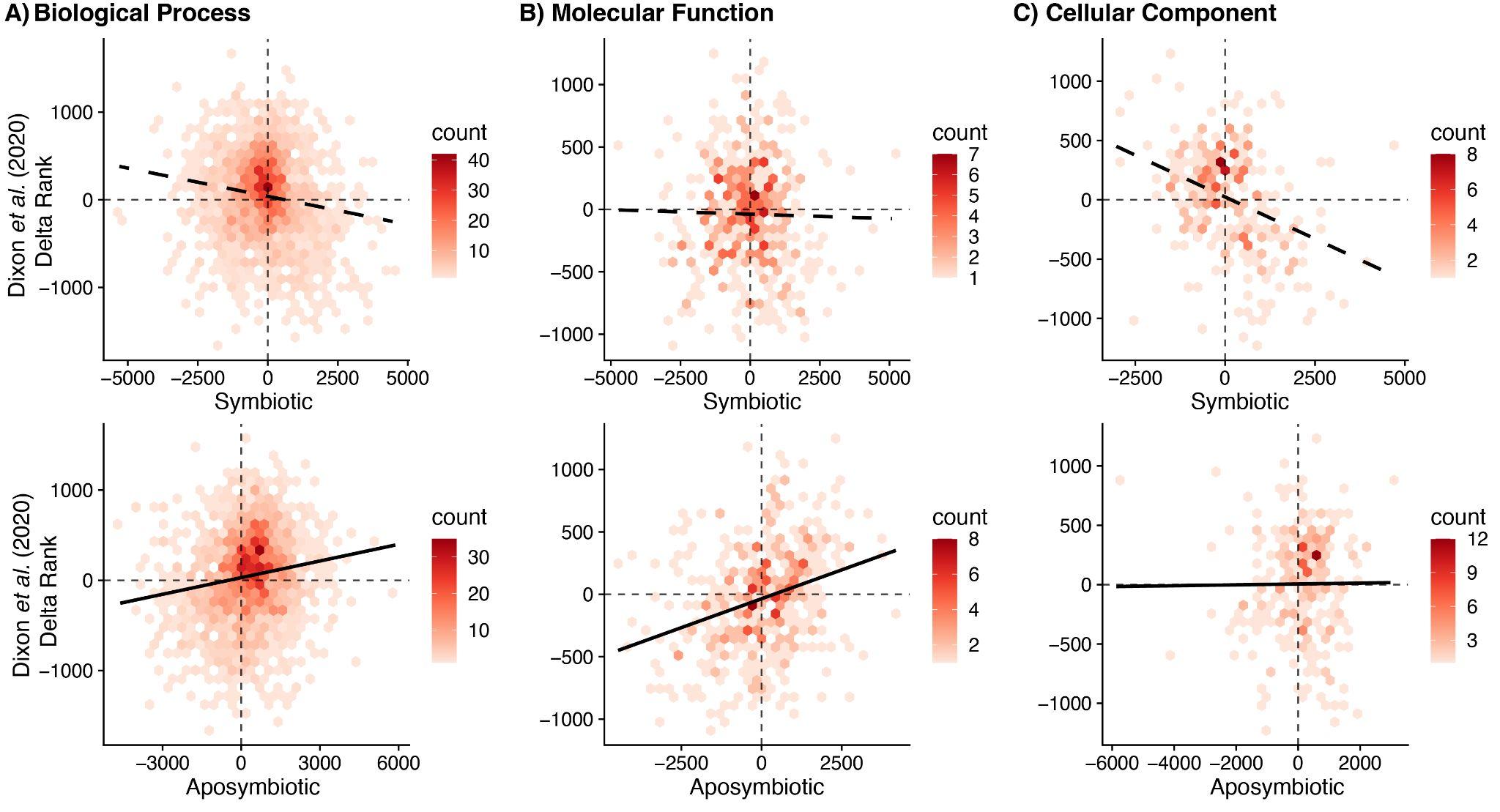


##### **Figure S8 |** Comparison of gene ontology (GO) delta ranks from environmental stress response studies featured in (G. Dixon et al., 2020) with heat challenge. A positive slope indicates that a coral is showing signatures of severe environmental stress response (ESR) whereas a negative slope is more characteristic of moderate ESR.


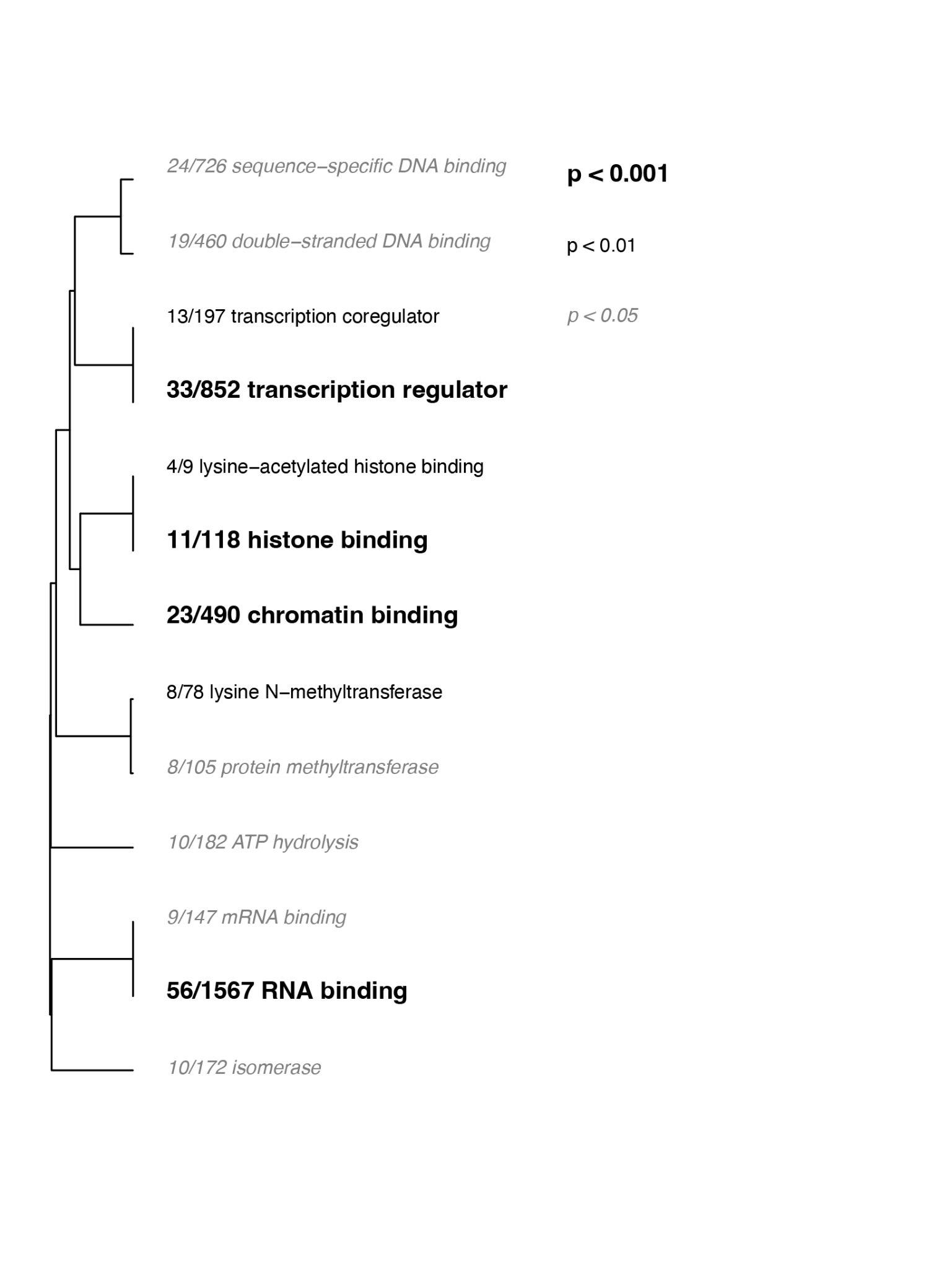


##### Figure S9 | GO enrichment results of the “molecular functions (MF)” category derived from the list of unique DEGs that responded to heat challenge in the symbiotic phenotype corals only. The dendrogram describes the relationship of shared genes between categories, and text size and boldness indicates the significance of each term.


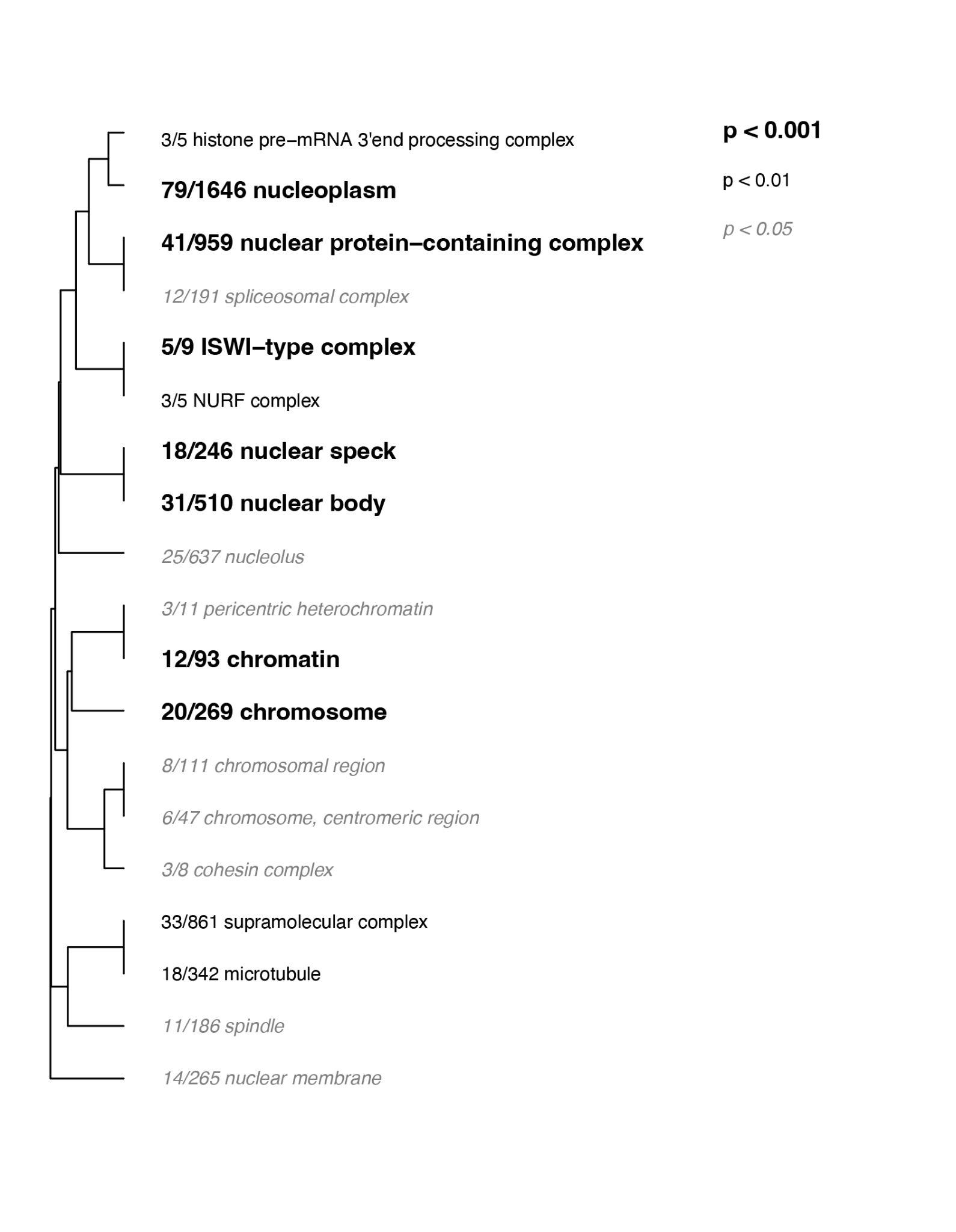


##### Figure S10 | GO enrichment results of the “cellular components (CC)” category derived from the list of unique DEGs that responded to heat challenge in the symbiotic phenotype corals only. The dendrogram describes the relationship of shared genes between categories, and text size and boldness indicate the significance of each term.
